# Supplementary material for: Multifunctionality of Nanosized Calcium Apatite Dual-Doped with Li+/Eu3+ Ions Related to Cell Culture Studies and Cytotoxicity Evaluation In Vitro
Source: Biomolecules. 2021 Sep 21;11(9):1388. doi: 10.3390/biom11091388 (PMC8466056; doi:10.3390/biom11091388)
Supplement: Supplementary file 1 [file biomolecules-11-01388-s001.zip › biomolecules-1336809-supplementary.pdf]

biomolecules

MDPI

Supplementary materials

Article

Multifunctionality of Nanosized Calcium Apatite Dual-Doped with Li^{+}/Eu^{3+} Ions Related to Cell Culture Studies and Cytotoxicity Evaluation *In Vitro*

Paulina Sobierajska[1,*], Blazej Pozniak[2], Marta Tikhomirov[2], Julia Miller[3], Lucyna Mrowczynska[4], Agata Piecuch[5], Justyna Rewak-Soroczynska[1], Agata Dorotkiewicz-Jach[6], Zuzanna Drulis-Kawa[6] and Rafal J. Wiglusz[1,*]

1. Institute of Low Temperature and Structure Research, Polish Academy of Sciences, Okolna 2, 50-422 Wroclaw, Poland; p.sobierajska@intibs.pl; j.rewak@intibs.pl; r.wiglusz@intibs.pl
2. Department of Pharmacology and Toxicology, Faculty of Veterinary Medicine, Wroclaw University of Environmental and Life Sciences, C. K. Norwida 31, 50-375 Wroclaw, Poland; blazej.pozniak@upwr.edu.pl; marta.tikhomirov@upwr.edu.pl
3. Department of Immunology, Pathophysiology and Veterinary Preventive Medicine, Faculty of Veterinary Medicine, Wroclaw University of Environmental and Life Sciences, C. K. Norwida 31, 50-375 Wroclaw, Poland; julia.miller@upwr.edu.pl
4. Department of Cell Biology, Faculty of Biology, Adam Mickiewicz University in Poznan, Uniwersytetu Poznanskiego 6, 61-614 Poznan, Poland; lumro@amu.edu.pl
5. Department of Mycology and Genetics, Wroclaw University, Przybyszewskiego 63, 51-148 Wroclaw, Poland; agata.piecuch@uwr.edu.pl
6. Department of Pathogen Biology and Immunology, Wroclaw University, Przybyszewskiego 63, 51-148 Wroclaw, Poland; agata.dorotkiewicz-jach@uwr.edu.pl; zuzanna.drulis-kawa@uwr.edu.pl

* Correspondence: p.sobierajska@intibs.pl (P.S.); r.wiglusz@intibs.pl (R.J.W.); Tel.: +48-071-3954-274 (P.S.); +48-071-3954-159 (R.J.W.)

Abstract: Li^{+}/Eu^{3+} dual-doped calcium apatite analogues were fabricated using a microwave stimulated hydrothermal technique. XRPD, FT-IR, micro-Raman spectroscopy, TEM, and SAED measurements indicated that obtained apatites are single phased, crystallize with a hexagonal structure, have similar morphology and nanometric size as well as show red luminescence. Lithium effectively modifies the local symmetry of optical active sites and thus affect the emission efficiency. Moreover, the hydrodynamic size and surface charge of the nanoparticles have been extensively studied. The protein adsorption (lysozyme, LSZ; bovine serum albumin, BSA) on the nanoparticle surface depended on the type of cationic dopant (Li^{+}, Eu^{3+}) and anionic group (OH^{-}, Cl^{-}, F^{-}) of the apatite matrix. Interaction with LSZ resulted in a positive zeta potential and the nanoparticles had the lowest hydrodynamic size in this protein medium. The cytotoxicity assessment was carried out on the human osteosarcoma cell line (U2OS), murine macrophages (J774.E), as well as human red blood cells (RBCs). The studied apatites were not cytotoxic to RBCs and J774.E cells, however, at higher concentrations of nanoparticles cytotoxicity was observed against U2OS cell line. No antimicrobial activity was detected against Gram-negative bacteria with one exception for *P. aeruginosa* treated with Li^{+}-doped fluorapatite.

Keywords: Nanoapatites; Eu^{3+} and Li^{+} ions; Rare earth ions; Photoluminescence; Cytotoxicity; *In vitro* cell culture studies; Protein corona, Antibacterial evaluation; Theranostics

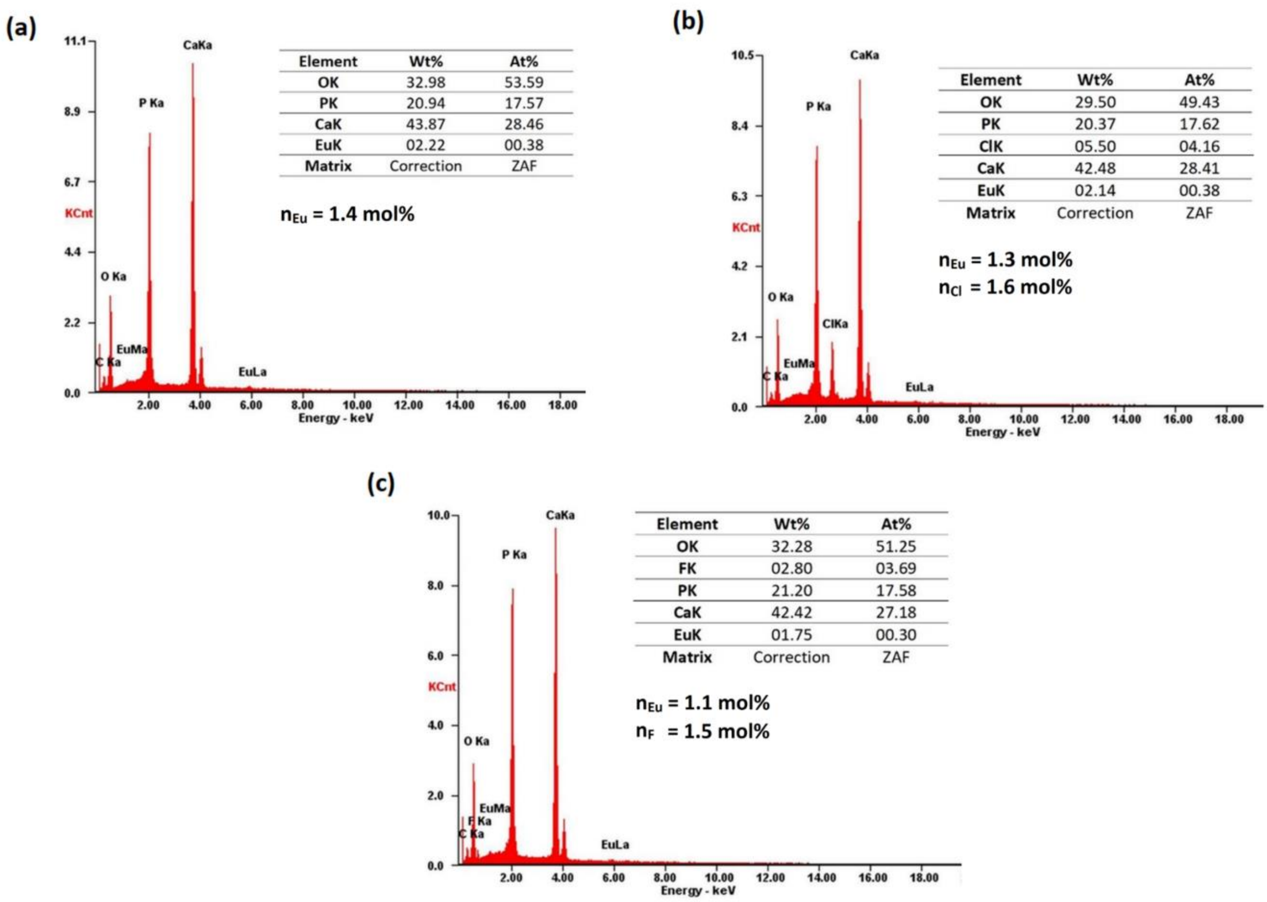

Figure S1: EDS spectra of the (**a**) $Ca_{10}(PO_4)_6(OH)_2$, (**b**) $Ca_{10}(PO_4)_6Cl_2$ and (**c**) $Ca_{10}(PO_4)_6F_2$ co-doped with 1 mol% Eu^{3+} and 2 mol% Li^+ ions, prepared at 500°C with the quantitative analysis of elements (inset).

Table S1. Lattice planes (hkl) assigned based on SAED images and ICSD data ($Ca_{10}(PO_4)_6(OH)_2$ (ICSD-26204), $Ca_{10}(PO_4)_6Cl_2$ (ICSD-24237) and $Ca_{10}(PO_4)_6F_2$ (ICSD-262707) of the $Ca_{10}(PO_4)_6(OH)_2$, $Ca_{10}(PO_4)_6Cl_2$ and $Ca_{10}(PO_4)_6F_2$ co-doped with 1 mol% Eu^{3+} and 2 mol% Li^+ ions, prepared at 500°C.

Sample	d (Å) SAED	(hkl)	d (Å) ICSD
$Ca_{10}(PO_4)_6(OH)_2$: 1 mol% Eu^{3+},2 mol% Li^+	3.4347	(002)	3.4395
	2.8253	(211)	2.8147
	2.2727	(310)	2.2636
	1.8690	(213)	1.8403
	1.3173	(431)	1.3169
$Ca_{10}(PO_4)_6Cl_2$: 1 mol% Eu^{3+},2 mol% Li^+	3.4231	(002)	3.4250
	2.7773	(112)	2.7801
	2.1901	(302)	2.1435
	1.8447	(213)	1.8418
$Ca_{10}(PO_4)_6F_2$: 1 mol% Eu^{3+},2 mol% Li^+	3.4270	(002)	3.4422
	2.7218	(300)	2.7041
	2.2419	(310)	2.2499
	1.8302	(213)	1.8372
	1.4555	(034)	1.4520
